# The Role of PERK in Understanding Development of Neurodegenerative Diseases

**DOI:** 10.3390/ijms22158146

**Published:** 2021-07-29

**Authors:** Garrett Dalton Smedley, Keenan E. Walker, Shauna H. Yuan

**Affiliations:** 1 Department of Neurology, University of Minnesota, Twin Cities, Minneapolis, MN 55455, USA; smedl009@umn.edu (G.D.S.); walk1189@umn.edu (K.E.W.); 2 GRECC, Minneapolis VA Health Care System, Minneapolis, MN 55417, USA

**Keywords:** unfolded protein response, endoplasmic reticulum stress, PERK, neurodegeneration, tauopathy, therapy

## Abstract

Neurodegenerative diseases are an ever-increasing problem for the rapidly aging population. Despite this, our understanding of how these neurodegenerative diseases develop and progress, is in most cases, rudimentary. Protein kinase RNA (PKR)-like ER kinase (PERK) comprises one of three unfolded protein response pathways in which cells attempt to manage cellular stress. However, because of its role in the cellular stress response and the far-reaching implications of this pathway, error within the PERK pathway has been shown to lead to a variety of pathologies. Genetic and clinical studies show a correlation between failure of the PERK pathway in neural cells and the development of neurodegeneration, but the wide array of methodology of these studies is presenting conflicting narratives about the role of PERK in these affected systems. Because of the connection between PERK and pathology, PERK has become a high value target of study for understanding neurodegenerative diseases and potentially how to treat them. Here, we present a review of the literature indexed in PubMed of the PERK pathway and some of the complexities involved in investigating the protein’s role in the development of neurodegenerative diseases as well as how it may act as a target for therapeutics.

## 1. Introduction

Neurodegenerative diseases are becoming a more pressing concern with the ever aging population [1]. The rapid advancements in genomics and molecular techniques have allowed progress in understanding how molecular pathways, and failures thereof, are responsible for the development of neurodegenerative diseases. A common feature among neurodegenerative disorders is the aggregation of misfolded proteins which lead to degradation and neuronal death (reviewed in [2,3,4]). Neurodegenerative diseases can further be characterized by the protein which accumulates leading to neurodegeneration. Alzheimer’s disease (AD), progressive supranuclear palsy (PSP), frontotemporal lobar dementia (FTLD), chronic traumatic encephalopathy, and primary age related tauopathy are all neurodegenerative disorders known as tauopathies defined by the misfolding and aggregation of hyperphosphorylated tau proteins [5,6,7,8,9] while other proteins, such as α-synuclein, prions, huntingtin lead to Parkinson’s, prior diseases, and Huntington’s disease, respectively [10,11,12,13] to name few. With the connection to protein malfunction and neurodegenerative diseases, protein maintenance and folding are primary targets for therapeutics.

Protein synthesis and folding are complicated processes leaving large room for error in how proteins are folded into their proper conformation. However, proteostasis is the process by which molecular pathways regulate the expression, proper folding, and correct localization of the proteome [14], and with a third of the proteome being synthesized within, the endoplasmic reticulum (ER) is a target of high value for this regulatory control. The primary pathway to ensure proteostasis within the ER is the unfolded protein response (UPR) (reviewed in [15,16]) where in the UPR attempts to refold the protein in order to prevent aggregation of misfolded proteins and reduce ER stress while managing other cellular processes to limit further stressors. However, methods of the UPR are not always successful.

When the regulatory pathway of the UPR fails, whether by malfunction of one of its components or outside influence, there are far reaching ramifications, one of which is disease. Using primary literature and published review articles indexed from PubMed, we examine and give comprehensive review the UPR pathway and its parts. Given its close connection to neurodegenerative diseases, we specifically highlight the PERK pathway according to the literature, as well as the contradictory, published data surrounding its function and investigate its potential as a target for developing therapeutics based on clinical and pharmaceutical trials. The literature reviewed include studies conducted in vitro, in vivo animal models and clinical retrospective studies. We used any article or review that suited the topic and report the topic to the best of our understanding.

## 2. Unfolded Protein Response Pathway and PERK

**Figure 1:**
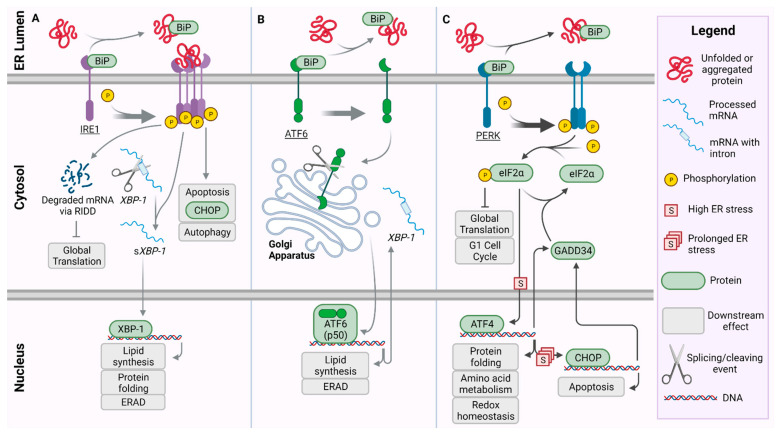
Pathways of the Unfolded Protein Response (UPR): In the presence of unfolded or aggregated protein, BiP dissociates from the three ER stress sensors: activating inositol requiring enzyme 1 (IRE1) (**A**), transcription factor 6 (ATF6) (**B**), and protein kinase RNA (PKR) like ER kinase (PERK) (**C**). The legend defines graphics for conserved structures between pathways of the UPR. (**A**) IRE1 forms a phosphorylated tetramer activating a cascade which promotes apoptosis via C/EBP homologous protein (CHOP) and autophagy. Simultaneously, IRE1 promotes the degradation of mRNA to reduce global translation via regulated IRE1-dependent decay (RIDD). The X-box-binding protein 1 (*XBP-1)* mRNA is additionally spliced, allowing XBP-1 protein to translocate to the nucleus and promote the transcription of UPR target genes for protein folding, lipid synthesis, and endoplasmic-reticulum-associated protein degradation (ERAD). (**B**) Following release of BiP and activation, ATF6 translocates to the Golgi apparatus where it is cleaved into p50-ATF6. p50-ATF6 serves as a transcription factor for target genes, including those involved in ERAD, lipid synthesis, and *XBP-1* which is translocated back to the cytosol. (**C**) PERK dimerizes and auto-phosphorylates, phosphorylating and inactivating eukaryotic initiation factor 2 alpha subunit (eIF2α). This reduces global translation and causes arrest of the G1 cell cycle via the adaptive UPR. Under high ER-stress conditions, activating transcription factor 4 (ATF4) is upregulated by phospho-eIF2α, then promoting transcription of target genes involved in protein folding, amino acid metabolism, and redox homeostasis. Over prolonged ER-stress, pro-apoptotic CHOP is activated. Accordingly, CHOP up-regulates GADD34, which in turn dephosphorylates eIF2α.

The UPR is composed of three pathways: inositol requiring enzyme 1 (IRE1), activating transcription factor 6 (ATF6), and protein kinase RNA (PKR)-like ER kinase (PERK) (Figure 1). In the event of ER stress, accumulating unfolded proteins bind directly to IRE1 serving as an activating ligand [17] (Figure 1A). The binding of the protein ligand causes IRE1 to homodimerize and autophosphorylate, allowing the serine/threonine kinase and an endoribonucelase domain in the cytoplasmic region to become active thus producing IRE1-α (Figure 1A). The activated IRE1-α functions in two capacities: it splices mRNA for the transcription factor X-box binding protein 1 (XBP-1) and activates an extension to the UPR termed IRE1-dependent decay (RIDD) which further limits global translation [18]. Once spliced (sXBP-1), sXBP-1 targets genes necessary for protein folding and degradation. This transcription factor helps lead to the upregulation of regulatory proteins involved in protein folding [19,20].

As the unfolded proteins accumulate, they also begin binding to binding immunoglobulin protein, (BiP/GRP78) causing BiP to release from ATF6 and PERK proteins and initiating these respective pathways (Figure 1B,C). BiP is a chaperone protein which natively binds to the luminal side of ATF6, IRE1, and PERK [21], and while it prevents activation of ATF6 and PERK, its binding to IRE1 has been shown to leave the regulatory levels of the IRE1 pathway unaffected [22]. Release of BiP from ATF6 reveals a localization signal allowing ATF6 to be transported to the Golgi apparatus via coat protein-II coated vesicles [23,24]. Within the Golgi apparatus, ATF6 is cleaved at the luminal domain by site-1 protease producing two halves. The N-terminus remains bound within the membrane but is later cleaved by site-2 protease. The cleavage events of ATF6 produces a bZip transcription factor, p50-ATF6, which is released from the membrane allowing it to be translocated to the nucleus [25,26,27]. In the nucleus, p50-ATF6 acts as a transcription factor for target genes, including endoplasmic-reticulum-associated protein degradation (ERAD) and XBP-1.

The PERK protein pathway is crucial in the management of the UPR and its activity in ER stress, and because of its many branching pathways, it has been implicated in the development of several diseases [28]. PERK is a transmembrane protein in which the BiP chaperone protein binds the luminal, N-terminus, while the cytosolic, C-terminus possesses the serine/threonine kinase domain [21]. BiP dissociates from PERK upon recognition of protein aggregation, at which point PERK homodimerizes (although there is some evidence suggesting it forms a tetramer [29]) and autophosphorylates. Phosphorylated PERK (PERK-P) now has an active kinase domain which phosphorylates the eukaryotic translation initiation factor 2 alpha (eIF2-α) [21,30]. The phosphorylation of eIF2α (eIF2α-P) shuts down protein synthesis and allows the ER to alleviate the source of the stress. Assuming the source of the ER stress has been managed, protein folding can resume and eIF2α-P is dephosphorylated, becoming inactive again; however, in instances in which the stress remains unresolved, the activating transcription factor 4 (ATF4) branch of the pathway is activated [31,32]. Functioning as a bZip transcription factor, ATF4 acts by binding to the cAMP response element (CRE) to regulate the expression of genes that play key roles in controlling redox homeostasis, protein folding, and amino acid metabolism [33,34]. This first attempt at stress relief is generally referred to as the pro-adaptive response; however, in instances of prolonged ER stress, ATF4 initiates a secondary pro-apoptotic signaling pathway by transcriptionally regulating the expression of growth arrest and DNA damage-inducible 34 (GADD34) and activating C/EBP homologous protein (CHOP/GADD153) [30,35,36,37,38]. GADD34 can then bind to protein phosphatase 1C (PP1C) to dephosphorylate and inactivate eIF2α restoring function of the protein synthesis pathways [39]. CHOP functions in tangent with ATF4 to increase the expression of GADD34, allowing translation to continue, furthering to increase ER stress in kind, and ultimately leading to cell death [40]. CHOP also down regulates B cell lymphoma-2, a notable anti-apoptotic protein family that functions to nullify proteins required for BAX/BAK dependent apoptosis, while also increasing the expression of pro-apoptotic proteins such as BIM [41,42,43]. Here, the PERK pathway downregulates cyclin D1 via eIF2α and causes G1 cell cycle arrest. This halts the processes required for cellular duplication allowing time for the cell to manage ER stress while reducing the necessity for resource-heavy metabolic pathways [44].

## 3. Crosstalk between PERK and Pathways of the UPR

Recent work is beginning to expand our understanding of the UPR by showing the high level of crosstalk between the ATF6, IRE1, and PERK pathways (reviewed in [45]), and more specifically, this work is showing the importance of PERK and its role in altering the trajectory of the ATF6 and IRE1 pathways. As mentioned, BiP is a target gene for ATF6α; however, in cells lacking functional PERK-dependent signaling, BiP was also found to be non-functional [38,46,47]. Additionally, although the mechanism is not yet completely understood, studies have also demonstrated from in vitro and in vivo models that functional PERK signaling, more specifically functional PERK, eIF2α, and ATF4, are necessary for the activation of the ATF6α pathway in the presence of ER stress [48,49]. ATF4 in particular appears to have an important role in the functional activity of the ATF6α pathway in that Teske et al. showed ATF4 was required for accurate transport of ATF6α from ER to Golgi apparatus during events of ER stress [49].

The influence of PERK extends beyond ATF6. In mouse embryonic fibroblasts with PERK knockout and eIF2α which was unable to be phosphorylated it was demonstrated that XBP-1 mRNA was not up-regulated during ER stress [50,51]. Production of XBP-1 is not completely dependent on the ATF6 pathway but was shown to be short lived in stressed conditions though stabilized by eIF2α-P and the resulting decrease in translation [52]. It has since been hypothesized that this stabilization would allow build-up of XBP-1 mRNA in preparation for the continuation of protein synthesis and thus increased levels of XBP-1 protein. These relationships are complicated due to the reach of each of the UPR pathways, and while different models present unique insight into these systems, variation in methods may lead to contradictory data. Nevertheless, the presence of interactions between PERK and adjacent UPR pathways of ATF6 and IRE1 are certain.

## 4. The Role of PERK in Neurodegeneration

Malfunction in the unfolded protein response has been strongly linked to many human diseases, and more specifically, malfunction in the PERK pathway; however, this connection appears to be both preventative and detrimental. As discussed, the activation of the PERK pathway is intended to reduce ER stress by halting protein synthesis and the metabolic functions of the cells to allow time to alleviate stress caused by misfolded proteins. However, given prolonged periods of cellular stress, eIF2α remains phosphorylated, preventing mRNA translation and protein synthesis for extended periods of time. Cells under these conditions are not undergoing the metabolic processes necessary to regulate homeostasis which can lead to loss of function or cell death. This in turn can lead to an array of clinical disorders depending on the cells in distress; when neural cells undergo prolonged ER stress, it leads to neurodegeneration [53].

Neurodegenerative diseases are a group of disorders characterized by the functional and/or structural loss of neurons, in most cases as a result of protein misfolding or aggregation [54,55]. Because of the post-mitotic nature of neurons, they are particularly vulnerable to cellular damage and stress, and thus rely even more heavily on the regulatory systems in place for the quality control of protein folding and the success of adaptive features such as the UPR. The etiology of neurodegenerative diseases could be environmental (thoroughly reviewed in [1]) or genetic [56,57], though these two are not mutually exclusive. Despite this, age has been shown to play a significant role in the development and progression of neurodegenerative diseases as a result of the inherent decline in ER fitness and the response of the UPR [58]. Evidence that the UPR has been activated in neurodegenerative diseases can be tracked, but the aggregation of misfolded proteins in the ER specifically is not a hallmark for most of these diseases and the connection to the accumulation of misfolded proteins and ER stress is not exactly self-explanatory [26]. This mixed with the variety of methodologies used in studies investigating UPR function in affected tissues with the extensive network of crosstalk between pathways creates a complicated narrative.

Neurodegenerative diseases that develop as a direct result from the accumulation of misfolded phosphorylated tau protein forming neurofibrillary tangles (NFT) are categorized as tauopathies [9]. Alzheimer’s disease (AD) is the most prevalent tauopathy characterized by the accumulation of phosphorylated tau protein in NFT and senile plaques composed of aggregates of amyloid-beta (Aβ) in affected patients [11,59,60,61]. Though produced in healthy as well as pathological conditions, excessive synthesis, or degradation of Aβ can lead to plaque formation. The majority of cases of early onset inherited or familial AD are due to mutations in the presenilin-1 gene, which produces an important protein in the proteolysis process, while the remainder of cases are the result of intracellular Aβ accumulation and its effect on UPR signaling [62]. One study using SK-N-SH neuroblastoma cells showed a decrease in BiP expression in AD patients [63] while other studies using brain tissue from affected patients showed increased BiP expression present in AD hippocampus and temporal cortex though these tissues remained morphologically healthy [64,65]. Introduction of Aβ to cortical neuron cultures showed no activation of the UPR or detection of ER stress markers despite evidence of apoptosis [66] leading the researchers to suggest that the events of cell death as a result of ER stress were independent of the UPR pathways. A separate set of studies investigating post-mortem brain tissue from affected patients showed increased PERK-P, IRE1-P, and eIF2α-P in AD neurons but were nearly absent in NFTs [64,65,67,68]. Human AD brain tissue as well as transgenic mice models showed the accumulation of phosphorylated tau protein reduced ERAD activity and activated the UPR by way of increased levels of PERK-P [69]. Another study of mouse models of AD also showed a similar pattern with raised levels of eIF2α-P and PERK-P [70]. Calcium (Ca^2+^) is an important regulator in cell survival and can lead to apoptosis through ER stress pathways when not in homeostasis. Disruption of Ca^2+^ concentrations lower the cells capacity to efficiently fold proteins leading to accumulation of misfolded proteins and thus ER stress [71]. It has been hypothesized that the disruption of Ca^2+^ homeostasis within the ER as a result of Aβ accumulation and internalization causes activation of the UPR [26,72].

Non-Alzheimer tauopathies including PSP, FTLD, Pick’s disease, and sporadic corticobasal degeneration similarly show a primary pathological inclusion of NFT formation resulting from phosphorylated tau while notably lacking an Aβ component. Due to the genetic link of the PERK protein and its etiology, PSP has become a primary model for understanding the relationship between the PERK pathway and disease and potentially treating said diseases. In PSP and FTLD, phosphorylated PERK-P, eIF2α-P, and IRE1-P have been observed in parts of the brain [54,73,74]. These studies show that, specifically within cells such as neurons or glia, a high level of phosphorylated tau is accompanied by increased UPR signaling. There are potentially conflicting studies investigating signaling in the PERK pathway in tissues from PSP affected brains. One study showed an increase in PERK activity in the pons, midbrain, and medulla with eIF2α showing increased activity in the brainstem [74] while another showed a decrease in PERK and eIF2α-P in the frontal cortex [75].

Other neurodegenerative diseases result from an accumulation of proteins other than tau leading to the loss of neurons and have also been linked to the UPR and PERK pathway. Parkinson’s disease (PD) is a neurodegenerative disorder characterized by the deposit of ubiquitinated α-synuclein protein that create Lewy bodies leading to loss of dopaminergic neurons from the substantia nigra pars compacta [10,11]. The largest risk factor in the development of PD is age [76,77], giving way to the accumulation of proteins in the neurons which has been shown to activate the UPR as evidenced by phosphorylation of PERK and eIF2α in dysfunctional neurons, both in vitro and in vivo [78,79,80]. Huntington’s disease (HD) results from the accumulation of the intracellular protein huntingtin in the striatum of the neurons causing an increase in the expression of BiP and CHOP in the parietal cortex [81] and an increase in BiP expression and phosphorylation of IRE1 in striatal tissues from HD mice brains [82]. In prion disease models, accumulation of prion protein caused decrease in protein synthesis due to prolonged eIF2α-P, ultimately leading to synaptic and neuronal loss [13]. Cortical samples from prion diseases have been shown to have increased levels of GRP58, GRP78, and GRP94; however, analysis of ER stress markers prove difficult due to the relatively long post-mortem delay of infectious tissues and the relatively short half-life of these respective markers [72,83].

Amyotrophic lateral sclerosis (ALS) patients show increased PERK, ATF6, and IRE1 signaling, with higher levels of CHOP and BiP in the spinal cord [84], and increases in downstream elements of the UPR such as ATF4, XBP-1, and GRP58 [85]. In addition, the motor-neurons of mice modeling familial ALS showed increased UPR proteins PERK and ATF4 as well as an increase in immunoreactivity for BiP [86]. As discussed, a wide array of neurodegenerative diseases show some level of UPR signaling often with an increase in activity from the PERK pathway. Additionally, with these studies in mind, as well as the amount of crosstalk between the pathways of the UPR that are being identified, it is becoming clearer the extent to which PERK plays a role in the UPR and ER stress response. However, though there is a correlation between neurodegenerative diseases and the PERK pathway, the complexity of PERK’s role is also making it difficult to understand causation.

## 5. Genetic Components of PERK Failure

**Figure 2:**
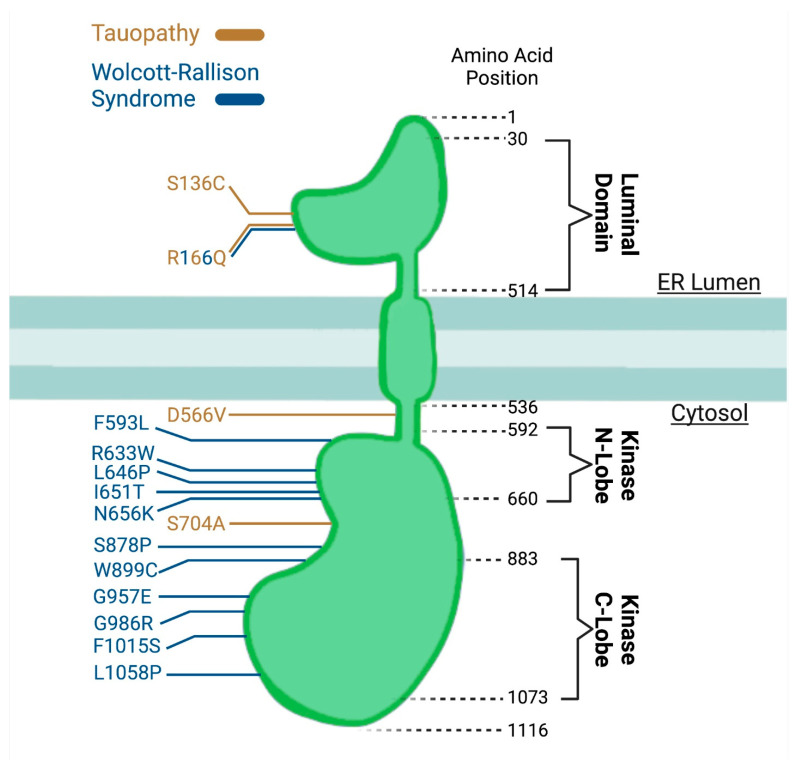
Pathology associated mutations of *EIF2AK3*/*PERK* protein. Functional regions of the protein are indicated by amino acid positions with dashed lines (black) and colored amino acid numbers indicate tauopathy-related PERK Haplotype B SNPs (gold) and Wolcott-Rallison Syndrome variants (blue).

Thus far, we have discussed the link between UPR signaling present in neurodegenerative diseases, but advancements in genetics are beginning to show how failure of PERK is leading to disease. Mice which have been genetically engineered with knockout of *Perk* develop early onset diabetes and have premature death [30,87]. In humans, the loss of function mutations in the *EIF2AK3* gene, which encodes PERK, cause Wolcott-Rallison syndrome (WRS) [88]. Wolcott-Rallison syndrome is a rare autosomal recessive pediatric disease in which patients present with neonatal diabetes, skeletal dysplasia, and growth retardation. The mutations can be organized into two categories: those which include non-sense mutations or frame-shift mutations, and ones which include missense mutations near the serine/threonine kinase domain. These mutations can occur throughout the gene (Figure 2), but a mutated PERK protein result in a loss of function [89] as shown from the fibroblasts derived from WRS patients that reported low or absent PERK activity during ER stress [88]. The disease severity is also potentially dependent on the level of PERK function given that disease onset and death are delayed in the missense mutations compared to the nonsense mutations suggesting that mutations clustering around the kinase domain may modify the activity of the protein to the point of loss of function of the PERK protein when compared to mutations elsewhere in the protein. Because WRS is exceedingly rare and likely under-diagnosed, our knowledge about it is limited and progress in understanding is slow. In addition to the cardinal clinical features, WRS patients can also have variable involvement in other organs, which leads to mental retardation, liver, heart, and renal failure. In examination of a post-mortem brain from a patient with WRS, AT8+ NFT was observed [90], suggesting that there are tauopathy related neurodegenerative changes due to loss of function of PERK.

Additional genetic evidence of possible PERK dysfunction associated with disease comes from broader human studies. Several genome-wide association study (GWAS) were completed for PSP [57,91,92]. One of the genes identified was *EIF2AK3* with the risk locus being in the intergenic region upstream of *EIF2AK3*. The polymorphism identified in *EIF2AK3* was also associated with an increased risk of late-onset AD when paired with apolipoprotein E (*APOE*) e4 allele [93]. Interestingly, this risk locus is associated with three additional single nucleotide polymorphisms (SNPs) in the coding region of *EIF2AK3* as well. These SNPs result in nonsynonymous mutations at amino acid position 136 from a serine to cysteine, at amino acid position 166 from an arginine to glutamine, and at amino acid position 704 from a serine to an alanine. Haplotype A includes the SNP combination of serine at 136 and 704 and an arginine at 166 and is considered non- or low-risk, while the combination of cysteine at 136, glutamine at 166, and an alanine at 704, described in the GWAS for PSP and tauopathy and were also observed in the GWAS analysis in bone, is considered risk haplotype (PERK B) [94]. Haplotype A (PERK A) are the most common occurrence of haplotypes, accounting for just under 70% of the population. PERK B is the second most common, accounting for about 30% of the population while the final haplotypes account for the remaining cases, less than once percent.

The functional consequences of the coding variants are unclear, and studies thus far have presented conflicting data. From these GWAS studies, an amino acid change at site 136 was predicted to be damaging and mutations at sites 166 and 704 were predicted to be benign [94]. One study showed that mutation of the *EIF2AK3* gene resulted in increased PERK activity [74]. However, using human induced pluripotent stem cells (iPSC) carrying the PERK B risk variant associated with tauopathies, we discovered that ER stress induced via tunicamycin showed that mutation of the *EIF2AK3* gene resulted in a hypomorph [56]. We found that PERK B tau protein levels were increased in homozygous risk variant cells compared to controls leading to an elevation in tau levels and neuronal death. These data suggest that the risk variant may affect normal tau metabolism. Perturbation of tau homeostatic levels could have pathological implications. Human AD brains have been found to have elevated levels of tau [95]. Tau becomes pathologic when it is mislocalized to the synapse [96,97], when it is hyperphosphorylated [98], when it is processed [99], when it interferes with nuclear transport [100], or when it is misfolded [101]. Dysregulation of tau level could make the cells vulnerable to development of any of these pathological states. Inconsistencies in the narrative of PERK’s role in tauopathies are, in part, due to the variety of methodologies. In addition, the ER stress response potentially varies dependent on the cell type; therefore, making it difficult to generalize experimental findings. Further investigation in the cell type specific response will illuminate if neurons or glia are more affected.

By exome sequencing, a rare variant in the *EIF2AK3* gene has been found to be associated with late-onset AD. The variant, p.R240H, was identified in a Dutch AD exome cohort and showed a trend toward association in the Rotterdam Study cohort [102]. Histological analyses showed PERK activation in the postmortem brain from the carriers of *EIF2AK3* p.R240H, similar to brains from AD patients, but not brains from normal controls. These findings are similar to previous reports that AD brains exhibit a higher level of PERK activation compared to the control [65]. However, it is not known how PERK activation and function are affected by the p.R240H mutation. These results contribute to growing evidence that genetic alterations are causing neurodegenerative diseases and help provide some additional clues as to how PERK dysfunction may lead to pathology.

Another genetic mutation involving the ER stress pathway is Wolfram Syndrome, which is a rare genetic disorder affecting children, that presents with onset of juvenile-onset diabetes, optic nerve atrophy, hearing loss, and neurodegeneration [103]. Wolfram Syndrome mainly affects the nervous system and the pancreatic islet cells due to their intense demand for secreted proteins. Two genes have been identified to cause Wolfram Syndrome. Mutations in *WFS1*, also the gene that codes for the protein known as wolframin an ER resident protein necessary for regulation of calcium levels, can be either autosomal dominant or recessive. Mutations in the *WFS1* gene cause loss of function and as a result lead to elevated ER stress and ER stress-associated cell death [104]. Although not as common as *WFS1*, mutations in *WFS2* or *CISD2* gene also cause Wolfram Syndrome. Additionally, found in the ER, *WFS2* regulates optimal UPR function. Under ER stress, *WFS1* mRNA levels are reduced in IRE1 and PERK knockout cells indicating WFS1 is regulated by IRE1 and PERK [104].

Vanishing white matter disease (VWM) is pediatric inherited leukoencephalopathy. Patients present with cerebellar ataxia and spasticity. Sometimes patients develop minor cognitive impairment, and provoking factors include febrile infections and head trauma [105]. VWM is caused by recessive mutations in the eukaryotic initiation factor 2B (eIF2B)-subunit genes, which include *eIF2B1* to *eIF2B5*, that functions as a guanine nucleotide exchange factor (GEF) for initiation factor eIF2. Forming a ternary complex within initiator-tRNA, eIF2-GTP initiates translation by scanning the mRNA for the start codon. When translation starts, eIF2 dissociates in an inactive GDP-bound form and recycles eIF2-GDP to eIF2-GTP, thereby reactivating it for the next round of translation. When PERK is activated during ER stress, eIF2B is sequestered by eIF2α-P in the integrated stress response, resulting in the downregulation of protein synthesis. Various stressors activate different kinases that phosphorylate eIF2α and lead to inhibition of eIF2B as a hetero-decameric complex containing two copies of all subunits. Mutations of VWM interfere with normal function by affecting the normal complex formation and stability and by binding to the substrate eIF2 and the GEF catalytic activity [106,107,108]. The disease can present at any stage of life with patients developing leukoencephalopathy after a stressful event, such as infection or trauma. The activation of the UPR causes selective damage to astrocytes and oligodendrocytes [106,109,110]. Integrated stress response inhibitor (ISRIB), a small molecule which activates eIF2B, has been shown to restore the catalytic activity to VWM mutant eIF2B [111]. In animal models of VWM, ISRIB improved brain white matter pathology and motor skills in mice with biallelic eIF2B missense mutations [112]. These findings further support that integrated stress response dysregulation is a central cause for VWM. How genetic diseases affect different branches of the UPR demonstrate the importance of regulating protein synthesis and the homeostatic control of the UPR and highlight to the potential of restoring the UPR to normal function could be a critical therapeutic approach.

## 6. Clinical Evidence of ER Stress Modulation as Treatment

The far reaching implications of the PERK pathway highlight its importance and its potential as a factor leading to neurodegenerative diseases, and because of this connection, PERK has also become a target for the basis for clinical treatments [4]. Trazodone, which acts as a PERK inhibitor by reducing the levels of ATF4, has been shown in tauopathy animal models to reverse the toxicity of tau overexpression [4]. These findings have fueled interest in trazodone as a treatment for tauopathy and have led to further clinical studies. However, several human studies have resulted in conflicting conclusions about trazodone as a treatment for tauopathies [113,114]. A large population-based study utilized the electronic health records in The Health Improvement Network (THIN), which archives anonymous medical and prescription records from primary care clinics in the United Kingdom, including records of over 15 million patients. The authors assessed 4596 users of trazadone and 22,980 users of antidepressants other than trazodone. They then compared the risk of dementia in patients who were prescribed trazodone versus other antidepressants. The median time to dementia diagnosis for people prescribed trazodone was 1.8 years compared to the 1.1 years for people prescribed an antidepressant other than trazodone. However, the authors concluded that there was no association to the reduction in dementia with the use of trazodone nor that trazodone presented a neuroprotective effect [113]. In contrast, a retrospective study examining trazodone’s effect on Alzheimer’s dementia as a slow wave sleep enhancer showed that the trazodone non-users had a 2.6 fold faster decline based on the mini-mental status exam, which is a commonly used memory test, compared to the trazodone users [114]. In further analysis of the data, the protective effect of trazodone was only true in patients who had baseline sleep disturbance, suggesting that the protection might be related to trazodone’s effect on sleep. The discrepancies between the trial results could be due to sampling and the mechanism of action of trazodone. A more carefully designed trial will help to determine if trazodone could be beneficial in treatment of AD. A clinical trial designed to test trazodone in dementia is ongoing in the United Kingdom, thus the results from this new trial may provide new evidence regarding trazodone’s use in dementia treatment.

In addition, recent reporting from a phase II double-blind placebo-controlled trial, the CENTAUR trial, shows the potential of targeting the ER stress pathway for the treatment of neurodegenerative diseases [115,116]. The CENTAUR trial tested the combination of sodium phenylbutyrate and taurursodiol in ALS patients. Sodium phenylbutyrate is a known ER stress modulator proposed to function as a histone deacetylase inhibitor by acting as a chemical chaperone and to upregulate heat shock proteins [117,118]. Taurursodiol, also known as tauroursodeoxycholic acid helps to prevents apoptosis due to its role in the BAX pathway by preventing translocation of the BAX protein into the mitochondria [119]. The trial showed slowing of functional decline and increased survival in ALS patients, but longer and larger trials are necessary to further verify the efficacy of this combination [115,116]. These results are very encouraging and support drug development for neurodegeneration by manipulating the ER stress pathway.

## 7. Discussion/Future Directions

The importance of PERK in the UPR pathway to manage cellular stress cannot be understated. The ever-increasing cases of neurodegenerative diseases make investigation into these neurodegenerative diseases, their cause, and treatment a high priority. The molecular mechanisms involved in how PERK maintains homeostasis are still being defined but identifying the connection between the malfunction in the PERK pathway to the development of neurodegenerative disorders is presenting certain challenges. Differences in methods are producing conflicting narratives and the complexity of the PERK pathway as well as the level of crosstalk between other UPR pathways are proving it difficult to understand the far-reaching implications of the PERK pathway.

Current bioinformatic workflows present the opportunity for detailed comparison of large genetic datasets. Comparing the genome or transcriptome from patients affected by a neurodegenerative disease to unaffected patients highlights genetic differences and further points of interest to understand how these genetic differences lead to the presented disorder. In the recent past, the GWAS study described highlighted SNPs found in PSP patients. These SNPs are currently being investigated by creating mutant cell lines using CRISPR-Cas9 technology to investigate how these mutations affect the function of the PERK pathway. While this serves as a good example of how we can study these disorders, the field of bioinformatics is advancing rapidly making the extensive sequencing of genomes and transcriptomes more readily available. In tandem with further advancements in genetic editing methods, we are presented with ever broadening possibilities of application to study neurodegenerative disorders and how to begin treating them.

## Data Availability

Not applicable.
